# Supplementary material for: Machine learning-based meta-analysis of colorectal cancer and inflammatory bowel disease
Source: PLoS One. 2023 Dec 22;18(12):e0290192. doi: 10.1371/journal.pone.0290192 (PMC10745176; doi:10.1371/journal.pone.0290192)
Supplement: S1 Table — ‘Cases’ are tumour samples, and ‘Controls’ are adjacent normal samples from the same patients. All samples are taken using the biopsy. The ‘# of probes’ column indicates the number of probe sets on the respective microarray platform. Each probe set generally corresponds to a unique gene. (DOCX) [file pone.0290192.s001.docx]

| **Dataset** | **# of Cases** | **# of Controls** | **Platform** | **Country/City or State** | **Usage** | **# of Probes** |
| --- | --- | --- | --- | --- | --- | --- |
| [GSE21510](https://www.ncbi.nlm.nih.gov/geo/query/acc.cgi?acc=GSE21510) | 123 | 25 | GPL570 | Japan/Tokyo | Training | 54675 |
| [GSE44076](https://www.ncbi.nlm.nih.gov/geo/query/acc.cgi?acc=GSE44076) | 98 | 98 | GPL13667 | Spain/Catalonia | Training | 49386 |
| [GSE44861](https://www.ncbi.nlm.nih.gov/geo/query/acc.cgi?acc=GSE44861) | 56 | 55 | GPL3921 | USA/MD | Training | 22277 |
| [GSE68468](https://www.ncbi.nlm.nih.gov/geo/query/acc.cgi?acc=GSE68468) | 186 | 55 | GPL96 | USA/MD | Training | 22283 |
| [GSE89287](https://www.ncbi.nlm.nih.gov/geo/query/acc.cgi?acc=GSE89287) | 46 | 17 | GPL4133 | Netherlands/Zuid-Holland | Training | 45015 |
| [GSE103512](https://www.ncbi.nlm.nih.gov/geo/query/acc.cgi?acc=GSE103512) | 57 | 12 | GPL13158 | USA/New York | Training | 54715 |
| [GSE25070](https://www.ncbi.nlm.nih.gov/geo/query/acc.cgi?acc=GSE25070) | 26 | 26 | GPL6883 | USA/CA | Validation | 24526 |
| [GSE38026](https://www.ncbi.nlm.nih.gov/geo/query/acc.cgi?acc=GSE38026) | 16 | 16 | GPL11532 | Germany/Kiel | Validation | 33257 |
| [GSE24514](https://www.ncbi.nlm.nih.gov/geo/query/acc.cgi?acc=GSE24514) | 34 | 15 | GPL96 | Finland/Helsinki | Validation | 22283 |
| [GSE39582](https://www.ncbi.nlm.nih.gov/geo/query/acc.cgi?acc=GSE39582) | 566 | 19 | GPL570 | France/Paris | Validation | 54675 |
| [GSE113513](https://www.ncbi.nlm.nih.gov/geo/query/acc.cgi?acc=GSE113513) | 14 | 14 | GPL15207 | China/Fujian | Validation | 49395 |
| [GSE41657](https://www.ncbi.nlm.nih.gov/geo/query/acc.cgi?acc=GSE41657) | 25 | 12 | GPL6480 | China/Beijing | Validation | 41076 |
| [GSE110225](https://www.ncbi.nlm.nih.gov/geo/query/acc.cgi?acc=GSE110225) | 17 | 17 | GPL96 | Greece/Athens | Validation | 22283 |
| [GSE13294](https://www.ncbi.nlm.nih.gov/geo/query/acc.cgi?acc=GSE13294) | 155 | 0 | GPL570 | Denmark/Aarhus N. | Validation | 54675 |
| [GSE13067](https://www.ncbi.nlm.nih.gov/geo/query/acc.cgi?acc=GSE13067) | 74 | 0 | GPL570 | Australia/Parkville | Validation | 54675 |
| [GSE14333](https://www.ncbi.nlm.nih.gov/geo/query/acc.cgi?acc=GSE14333) | 290 | 0 | GPL570 | Australia/Parkville | Validation | 54665 |
| [GSE17536](https://www.ncbi.nlm.nih.gov/geo/query/acc.cgi?acc=GSE17536) | 177 | 0 | GPL570 | USA/Nashville | Validation | 54675 |
| [GSE2109](https://www.ncbi.nlm.nih.gov/geo/query/acc.cgi?acc=GSE2109) | 351 | 0 | GPL570 | USA/Phoenix | Validation | 54675 |
| [GSE35896](https://www.ncbi.nlm.nih.gov/geo/query/acc.cgi?acc=GSE35896) | 62 | 0 | GPL570 | UK/Macclesfield | Validation | 54675 |
