# Supplementary material for: Machine learning-based meta-analysis of colorectal cancer and inflammatory bowel disease
Source: PLoS One. 2023 Dec 22;18(12):e0290192. doi: 10.1371/journal.pone.0290192 (PMC10745176; doi:10.1371/journal.pone.0290192)
Supplement: S2 Table — ‘Cases’ are inflamed samples, and ‘Controls’ are samples from healthy patients. All samples are taken using the biopsy. The ‘# of probes’ column indicates the number of probe sets on the respective microarray platform. Each probe set generally corresponds to a unique gene. (DOCX) [file pone.0290192.s002.docx]

| **Dataset** | **# of Cases** | **# of Controls** | **Platform** | **Country/City or State** | **Usage** | **# of Probes** |
| --- | --- | --- | --- | --- | --- | --- |
| [GSE16879](https://www.ncbi.nlm.nih.gov/geo/query/acc.cgi?acc=GSE16879) | 61 | 12 | GPL570 | Belgium/Leuven | Training | 54666 |
| [GSE22619](https://www.ncbi.nlm.nih.gov/geo/query/acc.cgi?acc=GSE22619) | 10 | 10 | GPL570 | Germany/Kiel | Training | 54675 |
| [GSE59071](https://www.ncbi.nlm.nih.gov/geo/query/acc.cgi?acc=GSE59071) | 82 | 11 | GPL6244 | Belgium/Leuven | Training | 33252 |
| [GSE102133](https://www.ncbi.nlm.nih.gov/geo/query/acc.cgi?acc=GSE102133) | 65 | 12 | GPL6244 | Belgium/Leuven | Training | 33252 |
| [GSE179285](https://www.ncbi.nlm.nih.gov/geo/query/acc.cgi?acc=GSE179285) | 70 | 31 | GPL6480 | USA/South San Francisco | Training | 41000 |
| [GSE9452](https://www.ncbi.nlm.nih.gov/geo/query/acc.cgi?acc=GSE9452) | 8 | 5 | GPL570 | Denmark/Copenhagen | Validation | 54675 |
| [GSE36807](https://www.ncbi.nlm.nih.gov/geo/query/acc.cgi?acc=GSE36807) | 28 | 7 | GPL570 | UK/London | Validation | 54675 |
| [GSE37283](https://www.ncbi.nlm.nih.gov/geo/query/acc.cgi?acc=GSE37283) | 11 | 5 | GPL13158 | USA/Chicago | Validation | 54613 |
| [GSE4183](https://www.ncbi.nlm.nih.gov/geo/query/acc.cgi?acc=GSE4183) | 15 | 8 | GPL570 | Hungary/Budapest | Validation | 54675 |
| [GSE48958](https://www.ncbi.nlm.nih.gov/geo/query/acc.cgi?acc=GSE48958) | 7 | 8 | GPL6244 | Belgium/Leuven | Validation | 33252 |
| [GSE92415](https://www.ncbi.nlm.nih.gov/geo/query/acc.cgi?acc=GSE92415) | 162 | 21 | GPL13158 | USA/Spring House | Validation | 54613 |
