# Supplementary material for: Machine learning-based meta-analysis of colorectal cancer and inflammatory bowel disease
Source: PLoS One. 2023 Dec 22;18(12):e0290192. doi: 10.1371/journal.pone.0290192 (PMC10745176; doi:10.1371/journal.pone.0290192)
Supplement: S3 Table — Orange cells are more prominent genes. (DOCX) [file pone.0290192.s003.docx]

| 1) TNS4 | 2) SLC7A5 | 3) HIST3H2A | 4) HGD | 5) SCD |
| --- | --- | --- | --- | --- |
| 6) GAPDH | 7) GPN1 | 8) COL21A1 | 9) SDF2L1 | 10) CRYAB |
| 11) TNFRSF11A | 12) RNF43 | 13) ATP6V0E2 | 14) CD1D | 15) GAL |
| 16) LRCH1 | 17) TCF20 | 18) L1CAM | 19) GRTP1 | 20) FMO5 |
| 21) PPFIBP1 | 22) SSFA2 | 23) SEMA3C | 24) C2orf49 | 25) DDX42 |
| 26) LXN | 27) NEK2 | 28) EZR | 29) CHP2 | 30) LRRC17 |
| 31) EIF4EBP1 | 32) ESM1 | 33) GMNN | 34) CD34 | 35) ZXDC |
| 36) KLF4 | 37) FRYL | 38) CCND1 | 39) C2 | 40) CD8A |
| 41) DONSON | 42) CRYBA1 | 43) SLC22A6 | 44) USP3 | 45) GZMK |
| 46) ZNF232 | 47) FAP | 48) OR51E2 | 49) MAPK9 | 50) SLC22A2 |
| 51) MELK | 52) DDX17 | 53) COL10A1 | 54) FA2H | 55) GFPT2 |
| 56) AGA | 57) PTPN3 | 58) ELF3 | 59) NUCKS1 | 60) PIM2 |
| 61) HAVCR1 | 62) HSD11B1 | 63) CHN2 | 64) TM4SF5 | 65) EFNA3 |
| 66) CCDC57 | 67) HOXA2 | 68) ENAH | 69) MAP2 | 70) NFE2L3 |
| 71) CHL1 | 72) EGLN3 | 73) TPH1 | 74) VAMP8 | 75) PNKP |
| 76) PMP22 | 77) ZNF264 | 78) ZNF239 | 79) LRMP | 80) TEX10 |
| 81) EDF1 | 82) IRF6 | 83) ARHGAP5 | 84) YTHDC1 | 85) SYT13 |
| 86) KRT81 | 87) CHFR | 88) NEDD4 | 89) LRRC2 | 90) BTNL8 |
| 91) DDX3Y | 92) SEZ6L2 | 93) LAS1L | 94) VPS4B | 95) NAV2 |
| 96) COG7 | 97) CXCL13 | 98) FAM65B | 99) TACC1 | 100) PFDN4 |
