# Supplementary material for: Machine learning-based meta-analysis of colorectal cancer and inflammatory bowel disease
Source: PLoS One. 2023 Dec 22;18(12):e0290192. doi: 10.1371/journal.pone.0290192 (PMC10745176; doi:10.1371/journal.pone.0290192)
Supplement: S4 Table — Orange cells are more prominent genes. (DOCX) [file pone.0290192.s004.docx]

| 1) HOXB2 | 2) DNAAF5 | 3) IRF7 | 4) TMEM39B | 5) C19orf25 |
| --- | --- | --- | --- | --- |
| 6) ABCA7 | 7) FXYD1 | 8) ZNF354A | 9) TRIM32 | 10) IGSF22 |
| 11) RNF122 | 12) SLC6A14 | 13) CIPC | 14) PDCD2L | 15) ABCB11 |
| 16) ADRA2C | 17) PLD2 | 18) PPCDC | 19) VEGFB | 20) LANCL3 |
| 21) LRRC56 | 22) ABLIM3 | 23) TXNDC16 | 24) BAZ2B | 25) YY1AP1 |
| 26) CCDC116 | 27) ATG2A | 28) IFI27L1 | 29) WBP2 | 30) TOX2 |
| 31) ZSCAN29 | 32) KDM4C | 33) GPATCH1 | 34) MARK2 | 35) MC5R |
| 36) ODF3L2 | 37) KIF22 | 38) DHX38 | 39) OSCP1 | 40) ZSCAN22 |
| 41) SYTL1 | 42) CDK7 | 43) SPATC1L | 44) JAG2 | 45) GLTSCR1L |
| 46) POLG | 47) DACT1 | 48) INPP5A | 49) SDF2L1 | 50) GUCA2A |
| 51) METTL1 | 52) REG4 | 53) APH1A | 54) TWF2 | 55) NGEF |
| 56) GCGR | 57) DNTTIP1 | 58) RBL2 | 59) TMEM268 | 60) ARMC5 |
| 61) TAT | 62) TSPAN11 | 63) TMEM87A | 64) SLC25A13 | 65) PSENEN |
| 66) DCUN1D3 | 67) MAPK14 | 68) CHRNA2 | 69) MOB2 | 70) RIF1 |
| 71) MPG | 72) CD300LF | 73) PRNT | 74) GUCY1A2 | 75) S100A7A |
| 76) GLYR1 | 77) CAPN5 | 78) APOA4 | 79) HLA-DQA1 | 80) DDX60 |
| 81) DSG2 | 82) SNAP29 | 83) PFN2 | 84) FEM1B | 85) UTP3 |
| 86) GIMAP4 | 87) DDC | 88) ACVRL1 | 89) TAGLN | 90) ALDOA |
| 91) ABHD3 | 92) TMEM41A | 93) VPS11 | 94) FBN1 | 95) WNT5A |
| 96) ST6GALNAC6 | 97) PTN | 98) C4BPB | 99) C2 | 100) HCK |
