# Supplementary material for: Machine learning-based meta-analysis of colorectal cancer and inflammatory bowel disease
Source: PLoS One. 2023 Dec 22;18(12):e0290192. doi: 10.1371/journal.pone.0290192 (PMC10745176; doi:10.1371/journal.pone.0290192)
Supplement: S5 Table — Orange cells are more prominent genes. (DOCX) [file pone.0290192.s005.docx]

| STAT1 | CCNE1 | PLK1 | STAT3 | BUB1 |
| --- | --- | --- | --- | --- |
| SMAD4 | MYC | ODF3L2 | RHEB | STAT6 |
| JUN | NRP1 | CDKN1B | TP53 | CCNA2 |
| CDKN1A | CCNA1 | AKT1 | ESR1 | POLR2A |
| RELA | MTOR | HIF1A | KAT2B | CDC20 |
| RHOA | CTNNB1 | XAB2 | EP300 | PTEN |
| AR | CCND3 | CENPE | AURKB | SMAD2 |
| HDAC2 | HDAC1 | CCNH | SKA1 | SLC3A2 |
| POLR2A | TRAF6 | SP1 | PCNA | EFTUD2 |
| LGR5 | CDKN2A | PRPF19 | MX1 | NOTCH1 |
| EPRS | TCF3 | SMAD3 | MDM2 | CCND2 |
| STAT5A | CDC5L | CHUK | KIF11 | SPC24 |
| ISG15 | IRF3 | ALDOA | RPTOR | CDT1 |
| CCNE2 | HSP90AA1 | GATA1 | SLC9A1 | CDK2 |
| NDC80 | RELASMAD4 | CDK6 | SLC9A3R1 | CREBBP |
| SPC25 | MNAT1 | MYD88 | CDK4 | TPI1 |
| GSK3B | CFTR | PPARA | NF2 | BUB1B |
| RB1 | COASY | YAP1 | CDK9 | ATF2 |
| NUF2 | SOX2 | IRF1 | MAML1 | SF3B1 |
| TTK | MAD2L1 | CASC5 | NR3C1 | - |
