# Supplementary figures and images for: Machine learning-based meta-analysis of colorectal cancer and inflammatory bowel disease
Source: PLoS One. 2023 Dec 22;18(12):e0290192. doi: 10.1371/journal.pone.0290192 (PMC10745176; doi:10.1371/journal.pone.0290192)

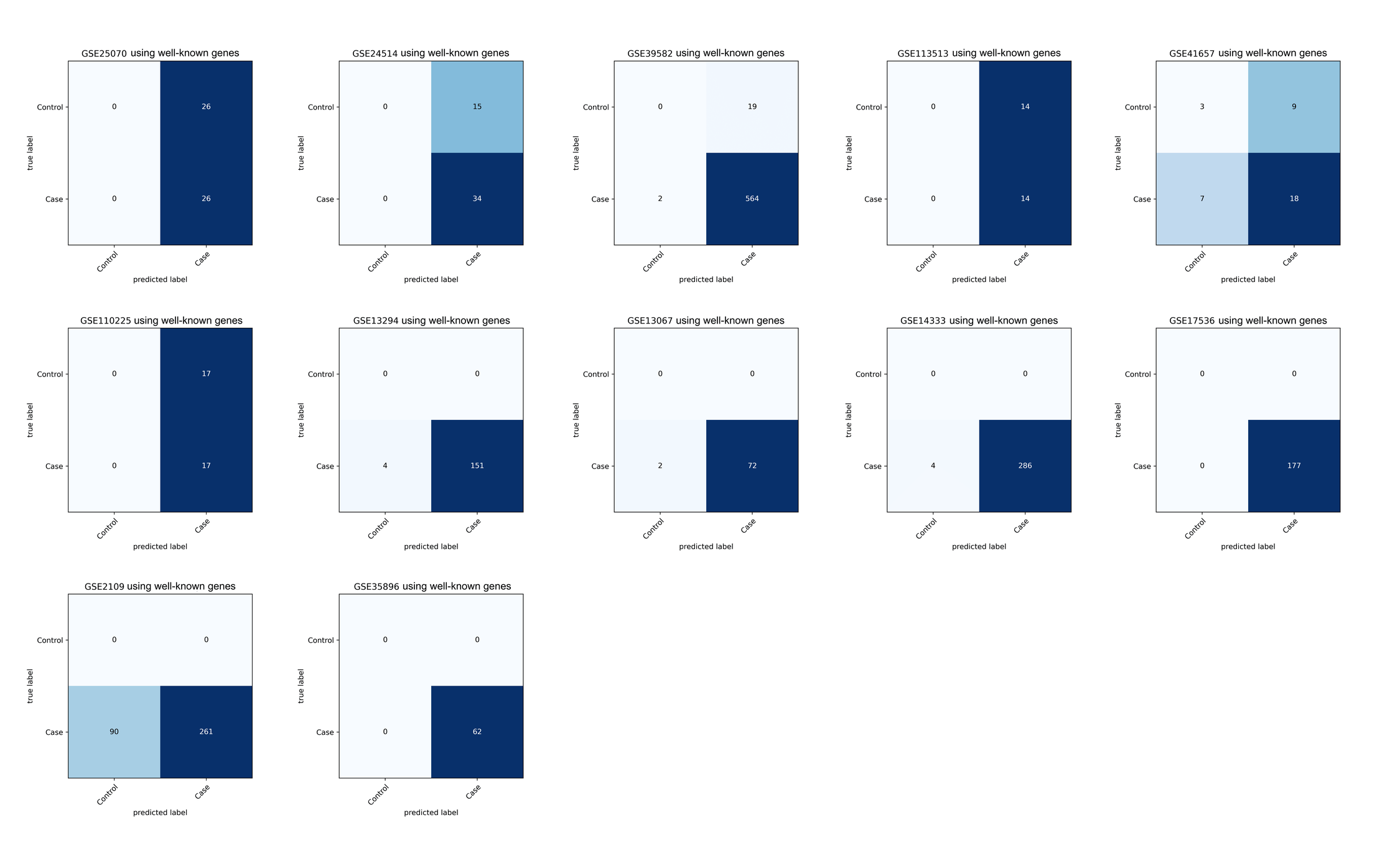

Supplement: S1 Fig — Training and validation were conducted using well-known genes: TP53, APC, KRAS, MGMT, SMAD2, and SMAD4. Confusion matrices are presented for the validation results. (TIF) [file pone.0290192.s006.tif]

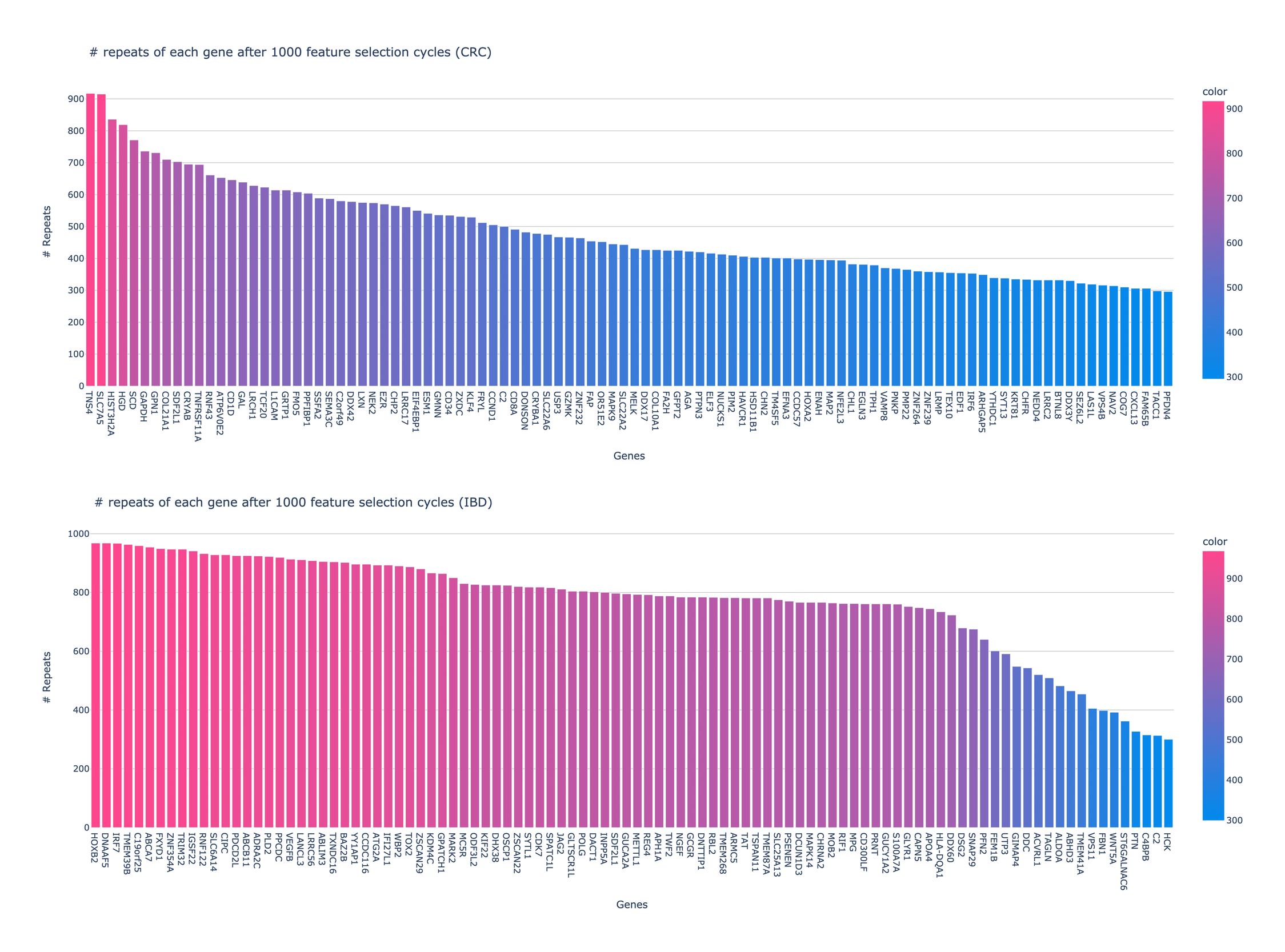

Supplement: S2 Fig — (TIF) [file pone.0290192.s007.tif]
